# Between principles and pragmatism – primary healthcare and social services professionals’ experiences and perceptions of self-care for older adults with home care: a qualitative study

**DOI:** 10.1080/02813432.2024.2389116

**Published:** 2024-08-09

**Authors:** Ing-Mari Dohrn, Åsa von Berens, Christina B. Olsson, Elisabeth Rydwik, Elin Jakobsson, Lina Palmlöf

**Affiliations:** a Stockholm Gerontology Research Center, Stockholm, Sweden; b Department of Neurobiology, Care Sciences and Society, Karolinska Institutet, Stockholm, Sweden; c Academic Primary Healthcare Centre, Region Stockholm, Stockholm, Sweden; d Medical Unit Occupational Therapy and Physical Therapy, Women’s Health and Allied Health Professionals Theme, Karolinska University Hospital, Solna, Sweden; e Department of Health Promoting Science, Sophiahemmet University, Stockholm, Sweden

**Keywords:** Focus groups, frail elderly, home care, preventive self-care, professional collaboration

## Abstract

**Objective:**

To explore the experiences of healthcare and social services professionals and their perceptions of using Certificate for self-care with support (CSS) for preventive self-care for older adults with home care, including the CSS process and collaborations between primary healthcare and social services.

**Design:**

An inductive qualitative study including seven focus group interviews analyzed with reflexive thematic analysis.

**Setting and subjects:**

The study was conducted in the Stockholm Region 2022/23. In total, 23 informants were recruited from four key partners involved in the CSS process: professionals from primary care rehabilitation and primary healthcare, social services officers, and home care staff.

**Result:**

The analyses resulted in five interconnected themes: ‘Guidelines with scope for interpretation,’ ‘Support for self-care is needed, but complicated in practice,’ ‘To trust the other professions’ competence,’ ‘There is a transfer of responsibility,’ and ‘Communication is key.’ The overarching theme ‘Principles or pragmatism for safe person-centered care,’ anchoring the other themes, revealed a common goal of achieving safe and individualized care within available resources, but from two conflicting perspectives: the importance of following the process according to the guidelines or taking a more pragmatic approach.

**Conclusion:**

This study highlights the need to establish structures facilitating safe self-care among frail groups, such as older persons dependent on home care. Our findings emphasize that the demarcation between, and responsibilities of, organizations need to be discussed and clarified to offer person-centered support. Comprehensible guidelines and functioning communication channels must be established so that all important perspectives can be heard, not least the patient’s.

## KEY POINTS

- The support of a patient’s self-care is an important part of health promotion and preventive efforts.
- Collaboration between social services and healthcare services is crucial when supporting an older person in performing their self-care.
- The boundaries and responsibilities of organizations regarding person-centered support for self-care need to be discussed and clarified.
- Clear guidelines and functioning communication channels must be established to ensure all important perspectives are heard, especially the patient’s.

## Introduction

The proportion of individuals aged 65 years or older in the EU is expected to increase from 21% in 2022 to 31% by 2100, with the proportion of those over 80 years old expected to more than double from 6% to 15% [1]. Sweden’s population is expected to follow this trend, impacting our society significantly and making it crucial to ensure that healthcare and social services are fit for purpose. To address these future challenges, enhanced collaboration between primary care and social services is needed, with an increased focus on health promotion, prevention, and rehabilitative efforts [2]. The importance of developing care models and support services tailored to the needs of older people with chronic conditions has also been emphasized [2].

An important part of health promotion and prevention is the support of self-care. Self-care can be defined as the ability of individuals, families, and communities to promote and maintain health, prevent disease, and cope with illness and disability, with or without the support of a healthcare provider [3]. It ensures continuity of care between interactions with the healthcare system, enabling individuals to manage their disease or disability and maintain well-being [4]. Self-care has also been described as a health resource that promotes self-responsibility as a part of healthcare, though not necessarily as an individualized practice; social support is an important prerequisite for effective self-care [5].

In Sweden, the National Board of Health and Welfare produces regulations and provides advice on how to comply with current legislation. The Board defines self-care as patients performing a healthcare intervention at home, either by themselves or with assistance, such as taking a prescribed medicine or doing exercises recommended by a physiotherapist [6,7].

Safely performing self-care in their own homes can be particularly challenging for older individuals, due to factors such as impaired hearing, vision, or cognitive decline [8]. The lack of comprehensive guidance on self-care practices for those reliant on home care assistance (i.e. social services provided in ordinary housing for those unable to meet their needs independently), combined with insufficient communication and collaboration among healthcare and social service providers, can exacerbate these challenges [2].

Nevertheless, it is essential to recognize self-care as a vital health resource for older individuals and tailor it to their abilities, functional limitations, and circumstantial constraints as much as possible [5]. Frail older individuals are at particularly high risk of impaired mobility and reliance on daily assistance [9]. Despite this, they can still benefit from preventive measures [10]. Therefore, providing adequate support and coordinating efforts to facilitate self-care for frail older persons are crucial to prevent further decline and enable prolonged residence in their own homes.

The healthcare services in Region Stockholm and the municipalities within the Region, responsible for social services including home care for older people, have formulated an agreement to clarify the roles and responsibilities of different partners regarding self-care, such as assessment, planning, and follow-up [11]. In the agreement, self-care is defined as a healthcare intervention that an individual can be responsible for and carry out, with or without practical help [11]. If an individual needs support to perform a self-care intervention at home safely, licensed healthcare professionals can, after assessing the patient’s health needs and possible risks, issue a Certificate for self-care with support (CSS) [6]. The CSS is used to apply for assistance for the patient from the social services (home care) [7].

Even though self-care is important to improve health and well-being, both from the perspectives of the healthcare services and the individual [4,6], recent data indicate that CSSs are rarely used for preventive or rehabilitative interventions [12]. A pilot project evaluating the feasibility of a collaborative working model between primary care and home care staff suggests that CSSs can be used for preventive self-care, such as home-based fall prevention training for older people [13]. However, the CSS process, including the communication structure and the assessment of the need for self-care, requires further investigation.

In this study, we used a qualitative research design to explore the experiences of healthcare and social services professionals and their perceptions of using CSSs for preventive self-care for older adults with home care, including the CSS process and collaborations between primary healthcare and social services.

## Methods

This was an inductive qualitative study using focus groups for data collection. Braun and Clarke’s method of reflexive thematic analysis was used to identify and analyze patterns in the data [14,15].

### Study setting, recruitment, and informants

The study was conducted in the Stockholm Region in 2022/23 as part of a larger project investigating the use of CSSs for health-enhancing interventions for older adults. According to the regional routines, when a CSS has been issued by a healthcare professional the older person must apply to the social services in the municipality to receive home care assistance with carrying out the self-care intervention. The goals and plan for the CSS should be person-centered and formulated in collaboration with the individual. Furthermore, if the person needs interventions from both healthcare and social services, a meeting to form a Coordinated Individual Plan (CIP) may be necessary [7].

We used purposeful sampling for the recruitment of informants with experience of CSSs to explore different perspectives regarding CSSs [16]. Professionals were recruited from four partners involved in the CSS-process: primary care rehabilitation professionals (physiotherapists (PT) and occupational therapists (OT)); primary healthcare professionals (general practitioner (GP), registered nurses (RN)); social services officers [biståndshandläggare], who perform a needs assessment and approve the provision of home care assistance, and home care staff. Informants were recruited through direct contact with managers at municipal and primary care centers, who in turn recruited relevant and interested employees. The inclusion criteria were having experience of either issuing CSS (PT, OT, GP, RN), determining the need for home care to support a patient with self-care according to CSS (social services officer), or experience of having been tasked with supporting individuals with their self-care interventions (home care staff). In total, 23 individuals were interviewed representing 11 of the 26 municipalities in the Stockholm Region. A short study-specific questionnaire was used to collect background characteristics, see Table 1.

**Table 1.** Informant characteristics.

	Primary care, rehabilitation (PT = 6, OT = 2)	Primary care, health care (GP = 1; RN = 3)	Social services officers	Home care service staff	Total
Women/men, *n*	7/1	4/0	4/1	6/0	21/2
Age, y	40 (28–59)	44 (35–48)	46 (43–61)	38 (34–47)	42 (28–61)
Time in practice, y	12 (2.5–27)	12 (5–15)	15.5 (3–25)	11.5 (7–20)	13 (2.5–27)
Time at current workplace, y	3 (1–8)	5.5 (2–7)	7 (3–22)	7.5 (3–12)	7 (1–22)
Issued^#^ CSS, *n*	2.5 (0–10)*	7.5 (1–10)	N/A	N/A	4 (0–10)
Processed^#^ CSS, *n*	N/A	N/A	5 (1–15)	N/A	5 (1–15)

Data are presented as numbers or median (range); *n* = numbers, *y* = years.

GP: general practitioner; OT: occupational therapist; PT: physiotherapist; RN: registered nurse; CSS: Certificate for self-care with support.

^#^Licensed healthcare professionals can issue a CSS if support to perform a self-care intervention is needed, and social services officers decide if additional home care will be approved, i.e. processing the CSS.

*One informant had not issued a CSS but had experience of supporting colleagues in the CSS process.

### Focus group interviews

Focus group interviews were chosen to take advantage of communicative interactions among informants sharing a common experience [16]. We conducted seven interviews: two with primary care rehabilitation professionals, two with primary healthcare professionals, one with social services officers, and two with home care staff. For logistical reasons, five interviews were conducted online using Microsoft Teams [17], while two face-to-face interviews with home care staff were held at their workplace. These interviews took place between December 2022 and March 2023, lasting 41–101 min, all moderated by the first author and assisted by the second author.

We used semi-structured interview guides with open-ended questions (Supplementary Table 1). The guides were developed by the first and second authors and reviewed and revised by the co-authors. The main domains addressed in the interview guides were experience of, and perspectives on, preventive self-care and the CSS process, including collaboration with the other key partners. Depending on the group being interviewed, the questions were added or adjusted to fit the interviewees specific experiences of CSS. The informants were encouraged to speak freely about their experiences and probing questions were used to obtain in-depth information. The interviews were audio-recorded and transcribed verbatim.

### Data analysis

We conducted a reflexive thematic analysis based on the six-phase analytical process outlined by Braun and Clarke [14,15]. The flexibility of thematic analysis offered the possibility for inductive analysis capturing both semantic and latent meanings in the informants’ narratives. Since the purpose of the study was to contribute to knowledge concerning experiences and perceptions of using CSSs for preventive self-care, we have been closer to an experiential than critical approach, while still acknowledging the social context. The analysis method enabled us to focus on the participants’ experiences through both descriptive and interpretative accounts of the data. The ‘Eight big tent’ criteria for qualitative research guided our process, from the design of the study to writing the manuscript [18].

Through the iterative reflexive process, it became clear that the initial codes for the different groups of professionals were semantically and latently coherent. As a result, all transcripts were analyzed as a single unit of analysis rather than separately by professional group. Theme development was predominately inductive, based on identifying and interpreting patterns of shared meanings. The process was recursive to ensure the themes were meaningfully coherent [14,15]. The analysis was performed in Swedish, and the results were then translated into English. See Table 2 for a more detailed description of the analytical steps.

**Table 2.** Description of the analysis based on the six-phase analytical process outlined by Braun and Clarke [11,12].

Phase	Description
1. Familiarization with data	Transcripts were read and re-read by the first and second author. The audio recordings were listened to simultaneously to gain a further contextual understanding of the data. Reflexive notes were made during the reading, listening, and coding.
2. Generating codes	The coding process was conducted by the first and second author. In the process the authors worked systematically through the entire dataset, and all items that could be useful in addressing the research question were coded. Both semantic and latent codes were used, and relevant items could be double-coded. The first and second author had continuous discussions through the coding process to create insight and increase understanding of data.
3. Developing themes	The codes were reviewed and discussed by the first and second authors. Discussion continued concerning the relationship between codes and initial meaning-based themes were generated. In this phase, codes were sorted into topic areas or clusters of meaning.
4. Reviewing themes	The team of co-authors repeatedly discussed findings, initial themes, and interpretations to confirm that the analysis remained true to the research question.
5. Defining and naming themes	The first and second authors revised the themes to create the most meaningful interpretation of the data. The writing of the manuscript began. The interviews were listened to once more by the first and second authors to try to ensure the relevance of the themes. Relevant names to describe the core of the themes were applied.
6. Writing the manuscript	Discussion continued between the co-authors about the themes and how to describe them. The writing of the manuscript was finalized.

During the process, the authors continually reflected on how their individual experiences and backgrounds influenced the assumptions and interpretations. All authors are healthcare professionals with clinical experience in primary care within a municipal setting, coupled with expertise in both quantitative and qualitative research. This influenced the choice of research questions and provided advantages in data collection and analysis, since a good understanding of the context contributes to a deep interpretation of the results. The study process has been underpinned by an assumption that knowledge and meaning are constructed through processes between what is already known and social discourse and experiences, i.e. a more constructivist than positivist view of what constitutes knowledge [19].

### Ethics

The study was approved by the Swedish Ethical Review Authority (Dnr 2022-04018-01) and all informants signed informed consent forms. Written information about the purpose of the study and the right to withdraw at any time without providing a reason was given to the informants upon recruitment and again verbally before the interviews.

## Results

The analyses resulted in five interconnected themes and a final overarching theme, presenting an umbrella concept anchoring the other themes.

### Theme 1 – Guidelines with scope for interpretation

It was clear that the concept of CSS was largely unknown and rarely used by primary healthcare professionals. The current legislation in Sweden and the regional guidelines gave scope for different interpretations, which led to uncertainties but also to some extent flexibility.

*It’s kind of slipped under my radar, you could say. (OT1)*

There was uncertainty about what type of activities should be considered as self-care, both among healthcare professionals and social services officers, and about the role of CSS versus the delegation of healthcare tasks. This uncertainty gave scope for different interpretations that could lead to frustration. As a result, local routines have been developed in some parts of the region. These local routines had not always been anchored with all the partners involved in the CSS process. The routines were described as providing guidance for the partners that had developed them but were, at the same time, perceived by other partners as making collaboration more difficult.

The role of CSS in incorporating rehabilitation into day-to-day home care was brought up by both rehabilitation professionals and social services officers. It was discussed that to promote and maximize independence, a person-centered approach is needed, shifting the focus from home care merely providing care towards one that supports and enables the older adults to participate in everyday activities. Currently, CSSs have not been used to facilitate this way of working, and questions were raised if this should be the case, given the tight schedules in today’s home care services. However, the common view was that a specific certificate should not be necessary for this to take place.

*Home care doesn’t have an everyday rehabilitation kind of mindset. There are some ‘diamonds’ who work hard and really want to make an effort with this, but it feels like the certificate shouldn’t exist for that. This is something that needs to change within the structure of the way the home care service thinks. (PT1)*

Reflections were also made on how CSSs were sometimes used to solve a problem or tackle a certain issue. It could be a way of getting routine healthcare tasks done without needing to synchronize home visits, e.g. when the older person did not want to give out their keys to more than one care provider or to create an increased sense of security for the older person.

*He’s a bit afraid of making a mistake [with the eye drops] and when they [home care staff] come, it simply gives him a sense of security, morning and evening. He gets the eye drops, and then he has someone to talk to and ask him how he is. (Social services officer 1)*

It could also be a way of avoiding time-consuming situations and simplifying the daily work.

*As for the socks… For compression class one, these must be included as clothing, but compression class two needs a certificate. But that can cause problems with the social services officer and then it’s sometimes easier to just write a certificate, even if it’s compression class one,… because you really can’t face having that discussion. (RN1)*

### Theme 2 – Support for self-care is needed, but complicated in practice

There was a strong consensus about the need for, and importance of, self-care in general, as well as the possibilities of supporting self-care for community-dwelling older adults receiving home care.

In particular, the PTs and OTs mentioned the need for life-long self-care, such as home-based exercise programs to prevent functional decline or the prevention of contractures for persons who have had a stroke. The resources allocated for primary care rehabilitation teams were mentioned and how these were restricted by time-limited treatment periods, which emphasized the need to support self-care.

*If the patient is lucky, they have relatives at home who can help… but in some cases there are no relatives and the patient lives alone, and we see that we could use the help of the home care service, by writing a certificate and instructing them how to carry out the intervention the patient needs for the rest of their life. (PT2)*

However, experiences of CSSs to facilitate continuous self-care, such as exercises at home or daily walking practice, also included uncertainties regarding the home care staff’s competence to support these interventions in a safe and correct way. This meant that even if a person applied for more home care hours to receive support for this type of self-care, which all partners considered necessary, the request might be denied by social services officers or home care services due to a cited lack of competence to support the individual safely. PTs and OTs also sometimes hesitated to issue a CSS due to doubts about the home care staff’s competence. Incorporating rehabilitation assistants into home care services to support these interventions was mentioned as a possible solution.

Another common and essential need for self-care highlighted was supporting older persons with taking their prescribed medication as directed. The frequency of CSSs being issued to support medication intake had recently increased because of changed guidelines that now prevented the delegation of medication administration to the home care staff. As a result, some primary healthcare units had recruited more assistant nurses to cope with the increased number of home visits related to medication administration, while others had chosen to use CSSs, which means that home care staff administer the medication. The visits from home care staff often coincided with the patient’s medication time, leading to discussions about the question of responsibility and competence for supporting medication administration, especially when the individual had a mild cognitive decline and needed to be reminded. The busy time schedule and low staff continuity in home care were also raised as major concerns.

### Theme 3 – To trust the other professions’ competence

Individual needs, and how they should be assessed and prioritized, was a topic raised by all participating partners. To be able to meet older persons’ needs and support a dignified life at home was a goal shared by everyone; however, differing perspectives did not always lead to the same decision. Having trust in other professions’ competence, assessments, and decisions was a core issue.

*Of course one trusts, in part, what the healthcare service has said and described, but it is not always correct. (Social services officer 2)*

The interviews revealed that the different partners had clear pictures of what their role was in assessing and meeting the individual’s needs in the CSS process but also ideas about what the other partners could and should contribute with. However, these ideas were not always shared by the other partners. Situations were described where the different professions’ specific views and competencies were not considered. This often led to frustration and missing opportunities to gain an overall picture, which hindered an effective person-centered process and the efficient use of resources.

*In my case, it was just that a cream should be applied and then it’s like: ‘Well, then a licensed doctor can take ten minutes to sit and write a certificate that the patient needs a cream to be applied’… It shouldn’t be something you need to have a certificate for. It just takes extra time, uses extra resources. (GP1)*

All partners considered that it is the home care staff who have the best insight into the individual’s overall situation and needs, at least if the staff have been employed for a long time and should therefore be more involved in decisions about self-care. The home care staff described how they sometimes needed to speak on behalf of the individual to express their needs, especially those who were frail and lacking support from relatives.

An important perspective to consider when assessing needs was the individuals’ own views of receiving support with their self-care. Challenges mentioned were their financial situation or their willingness to pay for more home care service hours, previous experience of medical or home care services and competences, or lack of insight into their own cognitive decline.

### Theme 4 – There is a transfer of responsibility

A major problem mentioned repeatedly by all partners was how the target group for CSS was described in the regional agreement and how the self-care interventions should be defined or interpreted. One of the crucial points highlighted in the interviews was that, according to the agreement, the patient must be able to take full responsibility for the intervention in the CSS. This was interpreted to mean that the older person should be capable of giving full instructions to the home care staff about how the self-care should be conducted and the support they need with it, and to actively ask for support without being reminded.

*I also think that the first thing to check when you are going to issue a certificate is whether the person really can take responsibility for all of this, and give instructions, if they understand? That’s probably the first box to tick on the paper they print out for the client. (Social services officer 3)*

This intentional or unintentional transfer of responsibilities due to the way things are done in practice was perceived as a problem. The roles and responsibilities of the different professionals were discussed, including responsibility for the intervention itself, the follow-up, and the transfer of information and instructions. An important question was how the responsibilities of different professionals could change in relation to the individual’s ability to perform their self-care.

*I think they shouldn’t just listen to the individual, but maybe also listen to those of us who are working with that patient. For five days in a row, he didn’t ask for his medicines, ask us to give them to him. Is it reasonable then that he is responsible for his medications? No, it isn’t. But his responsibility for the self-care continued, so you get a bit disconcerted. (Home care staff 1)*

Some social services officers described having to take responsibility and further assess the individual’s ability to manage their self-care with the support of home care staff before approving a CSS application. They referred to experiences of CSSs being issued by healthcare professionals without them, in their view, having an accurate picture of the individual’s abilities and home situation.

A more pragmatic view was presented by healthcare professionals, giving examples of CSSs being issued to patients in what they referred to as a ‘grey area’ concerning the individual’s ability to take full responsibility. These could be persons having some level of cognitive impairment where the healthcare professionals concluded that the self-care intervention, for example, intake of daily medicines, would work satisfactorily with support from experienced home care staff. They argued that a CSS could be justified to meet the needs of the individual and make the best use of available resources.

*There are staff who are superb … they have strong common sense, have worked for a long time and know their elderly patients and have a great relationship with them. They’re very solution-oriented and get in touch if they have questions or doubts and take excellent decisions. There is nothing complicated about giving medicines from a dosette box. So it’s ridiculous that that person should stand there, with all this ability, while another person has to come in [to give the medicines]. (RN2)*

From the home care staffs’ perspective, the ambiguous roles and responsibilities of self-care were perceived as a problem. Situations where they had to act outside their area of responsibility were described, e.g. when the older person forgot to remind the staff about the help they needed, or when they were asked to help with on-demand medications and there could be a risk of overdosing. They were also hesitant about being responsible for supporting an unsteady person with walking practice, or helping someone with eye drops when they felt they had not received sufficient instructions or lacked competence. They expressed concerns about being liable if something went wrong, even though it was the individual who formally had the responsibility for their self-care. This was described as a dilemma between adhering to their personal responsibility and wanting to help due to having the individual’s best interests in mind.

*Who is responsible then? If I give the wrong medication to a client … who is responsible? (Home care staff 2)*

The responsibility for follow up of CSSs was described as being at risk of ‘falling through the cracks’, especially if the patient had no contact with primary care but the need for support with self-care was ongoing with no time-limit. The staff from primary care were restricted by their time-limited treatment periods and expected the home care staff to contact them if the self-care was not working. However, the home care staff expected the person who issued the CSS to perform the follow up and experienced difficulties getting in contact with the healthcare professionals and social services officers to convey their views on how the self-care was working.

### Theme 5 – Communication is key

Communication with the other partners in the CSS process emerged as a recurring concern across all interviews and linked to the core issue addressed in all four previous themes. The importance of effective and well-functioning communication was repeatedly emphasized as either a solution or a prerequisite for being able to handle issues related to the interpretation of guidelines, responsibilities, assessment of needs, and optimal support for self-care. There was a great variation in the channels for communication in the CSS process, and these could sometimes depend on particular persons rather than routines. The consequences of poor or ineffective communication were described, but also situations where successful communication led to health benefits for the individual.

Communication between the healthcare professionals and the social services officer often only took place *via* the written CSS. Situations were described where CSSs were repeatedly issued, either given to the individual or sent directly to the social services officer, rejected with a comment from the social services officer, amended, and then sent back to the ‘issuer’. This delayed the process and created frustration among the partners involved, with a potentially negative impact on the individual’s wellbeing.

*There was a patient who had Parkinson’s, who didn’t dare exercise at home because of the risk of falling, so we initiated a certificate. The paper went back and forth and in the end it was the patient who said ‘no, let’s skip this shit’. Yes … then we put the whole thing on hold. (PT3)*

*We’re not trained in the official language that’s needed for writing these certificates. (AT2)*

At the same time, the social services officers revealed concerns about inadequately written CSSs and difficulties that arose when the intended intervention had not been communicated clearly enough to the individual.

*There have been some extremely incorrect certificates, incorrect assessments, and that is because of [the healthcare service’s lack of] resources. The patient has not received any information. They haven’t signed the certificate. We are working with this a lot. (Social services officer 2)*

Occasionally, the healthcare professionals deviated from the conventional CSS process to facilitate communication, for example, by contacting the home care service directly to ask them to support an individual with their self-care, i.e. not involving the social services officer.

Barriers to communication also related to the concepts or specific words used. A major concern presented by the PTs was that CSS applications were often rejected if the terms ‘training’, ‘exercise’ or ‘rehabilitation’ were used as a description of the desired intervention. These interventions were referred to by the social services officers as healthcare and therefore not something that could be included in a CSS. This was a problem that had been discussed in different group forums for PTs. Using alternative expressions, such as interventions for ‘preserving function’, was a way to get around the problem.

*You can twist everything around, you can make training not be training and you can make anything become training. Especially when it comes to rehabilitation for our stroke patients, I think. The smallest activity could be considered exercise. I mean putting the support socks on could be exercise. (OT1)*

All professions expressed that it would be easier if they could just sit down together and talk. A few examples were given when this had happened, mainly at CIP meetings, although these did not occur as frequently as required.

*I believe in having physical meetings, meetings in the patient’s home where everyone can be there and really see the problem for themselves and above all listen to the patient who is the most important actor in this. (PT1)*

### Overarching theme – Principles or pragmatism for safe person-centered care

The overarching theme revealed a common goal, which was to achieve safe individualized care in relation to the available resources, but from two conflicting perspectives: on the one hand, the importance of following the CSS process ‘by the book’ to ensure that responsibilities and professional boundaries were not violated and, on the other hand, the importance of solving the situation for the individual ‘here and now’, taking a more pragmatic view of the CSS process. Both perspectives were referred to as efforts to ensure a holistic view keeping the best interests of the older person in mind. Even though the terms ‘person-centered’ or ‘patient-centered’ care were not explicitly mentioned in the interviews, the perspectives discussed still aligned with the goals of these approaches. Both concepts aim to ensure that the care is individualized, respectful, and responsive to the person’s needs.

Obstacles along the way made it difficult to reach a shared understanding and offer safe person-centered support for self-care. These were, for example, limitations in the home care services agreements or difficulties in communication between the partners. Consequences of the transfer of responsibility highlighted the key question of patient safety and how, or if, it could be assured in relation to the different partners’ roles and boundaries, as well as the ability of the older persons themselves.

Even though the importance of self-care was not disputed, the applicability of the existing regional agreement was questioned by all partners. According to the ‘principles perspective’, the individual must be able to give clear instructions to the home care staff about how to perform the intervention without having to be reminded. As mentioned in the interviews, in reality, this would exclude a significant proportion of older persons who are dependent on help from both the healthcare and social care services, i.e. the target group for CSSs, unless a more pragmatic view was taken.

*I spoke to the social services officer last week and was told that the municipality rejects all certificates that are related to exercise or rehabilitation, since these are the responsibility of the healthcare service. They can approve eye drops sometimes, or most of the time in fact, but they refuse anything to do with rehabilitation or training. So now I don’t know when I’ll be able to write a certificate. That’s where I am right now. Not even if the patient is cognitively adequate or is able to communicate. They will anyway reject it, that’s the message we’ve been given. (PT2)*

## Discussion

The analysis revealed a common goal, which was to achieve safe and individualized care in relation to the available resources, but from two conflicting perspectives: the importance of following the CSS-process according to the guidelines or using a more pragmatic approach.

The concept of CSS was, to a large extent, unknown and rarely used by primary healthcare professionals, which was confirmed in a recent study [12]. The results reveal some possible explanations for this. One is that the process was described as complicated and that the issuers had experienced that the CSS ‘will anyway be rejected’ so they had given up. Other explanations were the unclear boundaries of responsibility for the intervention and patient safety concerns, including doubts about the home care staff’s competence and resources for supporting self-care.

It has been suggested that the boundaries of national guidelines can be lost in regional and local interpretations [20]. The local agreement gave scope for a narrower interpretation of the target group eligible for CSS than in the national guidelines based on current legislation, which limited the applicability significantly and made cooperation between the partners challenging. As a strategy to apply person-centered care for persons with cognitive decline, the home care staff had to adopt a more pragmatic approach, which was therefore not according to the regional agreement. This finding aligns with previous studies in dementia care [21]. Challenges in memory recall ability when learning how to perform self-care, as well as difficulty in knowing what to do when new situations or problems arise concerning their health condition, have also been described in relation to older adults without cognitive decline, which further emphasizes the importance of individualized support to achieve safe self-care [22].

The lack of efficient communication between partners in the process was a recurring issue in the results. Successful communication was described as being dependent on particular persons rather than the result of efficient routines. Something that further complicates communication and collaboration between the partners in the CSS process is that they work for different organizations: the Region of Stockholm (healthcare) and the municipality (social care services). It is well known that, in interprofessional collaboration, efficient communication is key to providing benefits for the individual clients or patients, and for making the best possible use of resources [23,24]. In interorganizational collaboration, it is particularly important to formalize the collaboration and to clarify and define roles, mandates, and responsibilities [23]. Our results revealed that this is needed in the CSS process.

Emphasis on the importance of applying a person-centered approach, within both healthcare and social services, has been highlighted in recent years. Person-centeredness means starting from, and focusing on, the individual person’s abilities, needs and circumstances in all parts of the care process [25,26]. The partners in the CSS process shared the goal of providing care with the best interests of the older person in mind. However, the group discussions often ended up focusing on difficulties regarding interprofessional and interorganizational collaboration. Different perspectives and competencies were not used as resources to achieve a person-centered care process, which created frustration among all partners involved.

In a person-centered care process with well-functioning collaboration, everyone involved, not least the older person, is given the opportunity to share and contribute with their perspectives and competences. Care professionals need to be responsive to the individual’s needs and desires; however, some individuals need support in expressing their needs [25]. This may especially apply to frail older people with cognitive decline who lack the support of relatives. Høy et al. [5] suggest that the critical issue is not who the care is provided by, such as a healthcare or social care professional, but whether the older person, as far as possible, is in control of the care and responsible for making their own choices.

Even though the high staff turnover in home care services is a major problem, the home care staff were, in general, considered to have the best knowledge about the individual’s situation. Home care staff may therefore be well placed to advocate for their client within the care network, although their ability to do so may be limited by their position within current routines and power structures. This has also been described among home care staff supporting people living with dementia [21,27].

It is known that preventive health-enhancing interventions can contribute to healthy and active aging with independence being maintained for a longer period and with costs for social services and healthcare needs postponed or avoided [28]. Providing self-care support for individuals living at home with home care is, therefore, an important task. However, a person-centered approach, appropriate competence, and well-functioning collaboration between the different partners is essential. When primary care resources are limited and providing support for continuous rehabilitation periods is not within the scope of social services’ responsibility or competence, there is an evident risk that older persons with needs will fall between the cracks and become overlooked.

### Methodological aspects

To ensure rigor and sincerity, we applied transparency in the presentation of study procedures and our backgrounds. Self-reflexivity has been a guiding principle that has been applied by the authors throughout the study process [18]. Rich descriptions, as well as quotations to elucidate the analytical claims, are included to strengthen credibility [14].

This study was carried out in a Research and Development environment as part of a larger research project aiming to produce knowledge that can be used in daily practice, which brings several strengths to the study. Among these are the broad expertise of the author group, a clinically relevant research topic that inspires commitment, and knowledge about the demand by professionals working both at a strategic and a clinical level for results from this type of project.

It is apparent that the interpretation of the local agreement between the Region and the municipalities had a great impact on the experiences and use of self-care, which may be seen as a limitation. Clearly, it is important to be cautious in drawing conclusions from a specific setting; specific contexts shape specific experiences and responses. Nevertheless, we believe that the results and insights from this study are also general in the sense that they can help develop understanding and be transferable to other contexts, which share important characteristics. Our study also contributes by adding to the body of literature, building a larger picture of the processes that are essential for supporting person-centered self-care.

Although experience with the CSS process was an inclusion criterion, some informants had limited exposure to it. During recruitment, finding informants with extensive CSS experience was challenging because it was rarely used. Nonetheless, all informants had significant experience working with community-dwelling older adults and addressing self-care support at home.

We chose to conduct five of the seven focus group interviews online since the informants were used to communicating via digital platforms and their workplaces were spread out over the Stockholm Region. Despite the practical considerations of the online format [17] and the small size of some groups (two informants), the participants interacted and were highly engaged during the interviews. While it can be debated whether a focus group can consist of only two persons, dyadic interviews are valuable. They offer the depth and detail of individual interviews while providing the interaction characteristic of focus groups [29]. The focus group interviews with the home care staff were held at their workplaces to accommodate their work schedules and due to uncertainty about their familiarity with digital platforms. From our point of view, these adaptations to the given context (mixing online and face-to-face interviews) enabled us to collect rich data purposeful for the study aim.

The interview groups were based on the informants’ professions, and thereby their common experience of supporting self-care, which facilitated interaction between individuals with similar perspectives. This probably impacted the results, as it allowed the informants to speak more freely about the other partners in the process, but also reinforced their own perspectives thereby missing opportunities for reflection in relation to others. It is known that meaning and understanding of phenomena can, to some extent, be constructed and reinforced by the social context [16].

We are aware that our own clinical backgrounds and current positions as researchers at a local research and development unit may have influenced how the informants shared their experiences, thoughts, and perspectives during the interviews. It is possible that the informants’ narratives were affected by ideas about how we could influence local stakeholders and decisions at strategic levels. We believe that this, most likely, provided us with richer material.

## Conclusions

This study highlights the need to establish structures facilitating safe self-care among frail groups, such as older persons dependent on home care. Our findings emphasize that the demarcation between, and responsibilities of, organizations needs to be discussed and clarified to offer person-centered support. There is an inherent complexity in writing guidelines that provide both good support but also scope for person-centered care, and our results show that issues about responsibility, target groups, and interventions applicable for the CSS need to be clarified. In addition to comprehensible guidelines, functioning channels of communication must be established so that all important perspectives can be heard, not least the patient’s.

## Supplementary Material

Supplementary table 1_Interview guide.docx
