# Supplementary material for: Between principles and pragmatism – primary healthcare and social services professionals’ experiences and perceptions of self-care for older adults with home care: a qualitative study
Source: Scand J Prim Health Care. 2024 Aug 9;43(1):36–46. doi: 10.1080/02813432.2024.2389116 (PMC11834817; doi:10.1080/02813432.2024.2389116)
Supplement: Supplementary table 1_Interview guide.docx [file IPRI_A_2389116_SM2204.docx]

| **Supplementary Table 1. Semi-structured interview guide** | |
| --- | --- |
| **Introduction** | Presentation of the researchers  Study aim  Short information about focus group interviews and practicalities  The interview is audio-recorded.  Ethical information |
| **Presentation of informants** | Name and workplace |
| **1. Tell us about your experiences of issuing/processing /supporting CSS** |  |
| Probing questions | When was this?  Tell us about the self-care interventions the CSS applied to.  Describe the patient (age, health condition, cognitive function etc.). |
| Primary care | What is central to your assessment of whether CSS is relevant?  Did you experience any practical obstacles when issuing a CSS? |
| Need assessors | What is central to your assessment of whether staff in the extended home care service should be approved to support self-care? |
| Home care professionals | How do you get information about the CSS and the support to be provided? |
| **2. In which cases has the CSS worked well/not so well? Why?** |  |
| Probing questions | What opportunities do you see for using CSS?  Challenges? |
| **3. Tell us about the collaboration with the other partners in the CSS process** |  |
| Probing questions | Which other persons are involved in the process?  What are their roles?  How does it work?  Tell us about the follow-up of a CSS |
| **4. We are aware that the CSS is rarely used; what would it take to increase the usage from your perspective?** |  |
| **5. How could the CSS be used for more preventive healthcare interventions?** |  |
| **6. Is there anything else about self-care, the certificate, or the process that you would like to add?** |  |
| General probing questions | Is there anyone who has had a different experience?  Does anyone recognize this?  Can you give an example? |
| CSS = Certificate for self-care with support |  |
